# Prevalence of *Leishmania* infection in refugee camps: A serological and molecular study in Gambella and Benishangul-Gumuz, Ethiopia

**DOI:** 10.1371/journal.pntd.0013280

**Published:** 2025-07-08

**Authors:** Habtamu Belay, Adugna Abera, Esayas Aklilu, Bortola Abdisa, Mahlet Belachew, Heven Sime, Myrthe Pareyn, Tesfahun Bishaw, Saskia van Henten, Johan van Griensven, Geremew Tasew, Berhanu Erko

**Affiliations:** 1 Ethiopian Public Health Institute, Malaria and Neglected Tropical Diseases Research Directorate, Addis Ababa, Ethiopia; 2 Aklilu Lemma Health Research Institute, Addis Ababa University, Addis Ababa, Ethiopia; 3 Public Health Emergency Management, Ethiopian Public Health Institute, Addis Ababa, Ethiopia; 4 Unit of Neglected Tropical Diseases, Department of Clinical Sciences, Institute of Tropical Medicine, Antwerp, Belgium; 5 Federal Ministry of Health, Disease Prevention and Control Directorate, Addis Ababa, Ethiopia; Institut Pasteur, FRANCE

## Abstract

**Background:**

Leishmaniasis, transmitted by sandflies, causes a severe health threat in East African refugee camps. High mobility, poor conditions and limited healthcare access heighten the risk of visceral leishmaniasis (VL) among refugees. Though, data on infection prevalence is remains limited. This study aims to determine the prevalence of *Leishmania* infection in refugee camps in Benishangul Gumuz and Gambella regions to improve VL detection and guide effective control strategies in humanitarian settings.

**Methods:**

A cross-sectional study was conducted from May to August 2023 in four refugee camps to determine *Leishmania* infection using DAT and rtPCR on blood samples. Sociodemographic and clinical data were collected using structured questionnaires. Ethical approval was granted, and informed consent was obtained. Data were analyzed using SPSS v23, with associations assessed using logistic regression and Chi-square tests at a 0.05 significance level. Continuous variables summarized by median and interquartile range (IQR).

**Result:**

The study included 1,223 participants (440 from Tsore camp and 220 from Sherkole in Benishangul Gumuz Region; 288 from Kule camp and 275 from Terkidi in Gambella Region), most of whom were from South Sudan (66.7%) and the majority were females (56.5%). 17.8% of the participants reported fever, with no spleen or liver enlargement and 0.2% lymph node swelling. Real-time PCR positivity was significantly higher in Tsore (14.6%, χ² = 21.4, p < 0.001), no significant difference in DAT positivity was observed across refugee camps (χ² = 6.6, p = 0.084). *Leishmania* DAT positivity rates were 6.0%, 4.6% and 4.7% in those with fever, chills and headache, respectively. *Leishmania* kDNA based rtPCR positivity rate were 11.7%, 8.8%, 7.3% and 6.2% in those with fever, chills, headache and weakness, respectively. Participants from Benishangul Gumuz region [AOR: 3.67 (95%CI: 1.57-8.59); p = 0.003]; South Sudanese [AOR: 2.87 (95%CI: 1.26-6.50); p = 0.012 and those with fever [AOR: 2.08 (95%CI: 1.01-4.28); p = 0.047] had a higher odds of DAT positivity. On the other hand, lower rtPCR positivity rates were seen in the Sherkole refugee camp compared to Tsore camps in Benishangul Gumuz region [AOR: 0.19 (95%CI: 0.08-0.45); p < 0.0001].

**Conclusion:**

*Leishmania* infection was prevalent in refugee camps in Gambella and Benishangul Gumuz regions. Asymptomatic cases and low parasite loads were common, highlighting the need for active case detection, intervention including treatment and vector control to manage VL transmission effectively.

## Author summary

Leishmaniasis poses a significant health threat in East African refugee camps, particularly among populations with high mobility, poor living conditions, and limited healthcare access. This cross-sectional study, conducted from May to August 2023, aimed to assess the prevalence of *Leishmania* infection in four refugee camps in the Benishangul Gumuz and Gambella regions. Using direct agglutination tests (DAT) and RT-PCR on blood samples from 1,223 participants, the study revealed a *Leishmania* infection prevalence of 4.0% by DAT and 10.3% by RT-PCR. Notably, RT-PCR positivity was significantly higher in Tsore camp (14.6%) compared to Sherkole camp (3.2%), where residents were 81% less likely to be tested positive. Fever, chills, and headache were common symptoms reported by the participants in varying proportions. Sociodemographic factors, such as being from the Benishangul Gumuz region or South Sudan, increased the odds of DAT positivity. These findings underscore the need for active case detection, intervention including treatment tailored interventions, including active case detection and vector control to manage visceral leishmaniasis (VL) in these vulnerable populations effectively.

## Introduction

Leishmaniasis is a parasitic disease caused by protozoan parasites of the genus *Leishmania*, transmitted through the bites of infected female Phlebotomine sandflies [1]. The disease has three clinical forms: Visceral leishmaniasis (VL) also known as kala-azar, which affects internal organs and is fatal if untreated; cutaneous leishmaniasis (CL), the most common form, causes skin ulcers; and mucocutaneous leishmaniasis (MCL) manifesting with both skin and mucosal lesions [2]. Leishmaniasis is recognized by the World Health Organization (WHO) as one of the neglected tropical diseases (NTDs) that significantly affects health in poor, in remote regions with limited access to healthcare [3].

VL poses a significant public health challenge in East Africa, where the region experiences some of the highest case rates globally, leading to outbreaks [4–7]. Factors believed to contribute to the occurrence of *Leishmania* infection include environmental changes, population movements between endemic and non-endemic areas, the presence of therapy-resistant strains, and immune suppression, which is often linked to malnutrition and co-infection with the human immunodeficiency virus (HIV) [4,8].

Population displacement plays a central role in the spread and transmission of infectious diseases, such as VL. The United Nations High Commissioner for Refugees (UNHCR) reported that there are more than 122 million displaced populations of these 37.4 million people are refugees across the globe in 2023 [9]. Refugees facing high mobility, poor living conditions and barriers to accessing health care are particularly susceptible to infectious diseases, with VL occasionally being identified in refugee camps [10]. In Kenya, an outbreak of VL was investigated in refugee camps in 2017. Thirty-four probable and confirmed VL cases were identified, with a case-fatality of 29.4%; in one case, molecular typing confirmed *Leishmania donovani* [5]. Moreover, reports revealed a strong relationship between civil unrest and VL [11].

The government of Ethiopia opened its borders for refugees, the majority originating from South Sudan, Somalia, Eritrea and Sudan. Ethiopia hosts a large number of refugees, many of whom are located in camps in the Gambella and Benishangul Gumuz regions, situated in the western part of the country. Large number of refugees in these camps came from areas where VL is endemic. Moreover, Gambella and Benishangul Gumuz regions have been identified as at high risk of VL based on geographical and climatological features [12,13]. Consequently, refugees could be at high risk of contracting and transmitting *Leishmania* parasites to the indigenous community and vice versa [14]. As *Leishmania* infections are often asymptomatic and the disease is difficult to diagnose, transmission can remain undetected for long periods of time.

VL is a public health concern in Ethiopia, particularly in vulnerable populations such as refugees. However, data on VL distribution in these populations is extremely limited. Refugee camps often lack the infrastructure needed for effective disease detection and control, and infections can spread rapidly in such settings. Timely identification of *Leishmania* cases can improve response efforts and reduce the disease burden. There is also a need for more global knowledge on managing VL in humanitarian and crisis settings. Thus, this study aimed to determine the prevalence of *Leishmania* infection among residents of the four refugee camps selected for the present study within Gambella and Benishangul Gumuz regions using serological and molecular analysis.

## Methods and materials

### Ethical statement

The study was conducted in accordance with ethical guidelines and received approval from the Aklilu Lemma Health Research Institute-Institutional Research Ethics Review Committee (ALIPB-IRERC) prior to data collection (Ref. No.: ALIPB IRERC/112/2015/23). A permission letter was obtained from Federal Democratic Republic of Ethiopia Refugees and Returnees Service (FDRE RRS) and subsequently from RRS regional office and camp coordination offices. Written informed consent or assent was obtained from all participants or, when necessary, from parents or guardians, with trained personnel assisting in participants’ native languages to ensure comprehension. Privacy and confidentiality were strictly maintained, with all identifying data anonymized and securely stored, accessible only to the research team. The study followed all relevant guidelines for research on human subjects, implementing additional safeguards to protect participants’ rights, dignity, and welfare.

### Study sites

**Fig 1:**
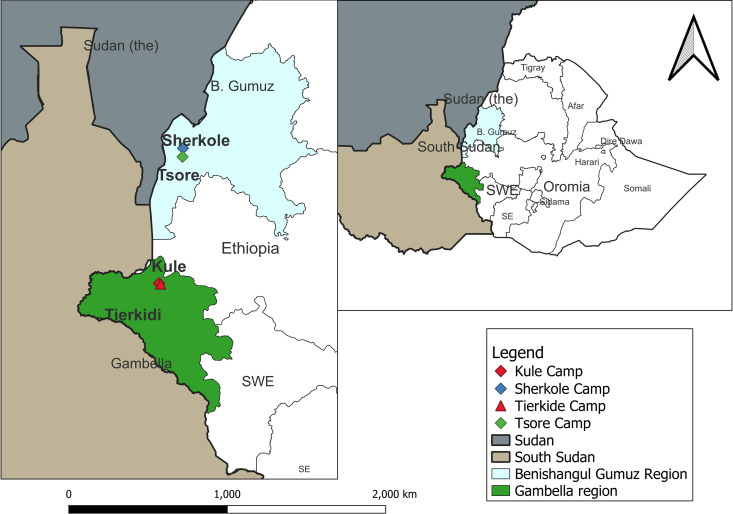
Map showing study refugee camps in Gambella and Benishangul Gumuz regions, Ethiopia [Kule Camp: represented by a blue diamond; Sherkole Camp: represented by a red diamond; Terkide Camp: represented by a red triangle; Tsore Camp: represented by a green square; Sudan: represented by a light gray color; South Sudan: represented by a dark gray color; Benishangul Gumuz Region: represented by a light blue color and Gambella Region: represented by a dark green color]. The maps were built using the free and open source QGIS software version 3.36.3 (QGIS Development Team (2024). QGIS Geographic Information System, version 3.36.3. Open-Source Geospatial Foundation Project. https://qgis.org) and shapefiles were obtained from the free and open-source site https://data.humdata.org.

There are a total of seven refugee camps in Gambella [15] and five in Benishangul Gumuz Regional States [16]. The four refugee camps selected for the present study are located in Itang and Homosha districts of Gambella and Benishangul Gumuz regional states, respectively (Fig 1). Itang, west of Gambella city, accommodates the Terkidi and Kule camps, sheltering thousands of South Sudanese refugees who fled violence since 2013 [15]. Similarly, Homosha, located near Assosa in Benishangul-Gumuz, is home to the Tsore and Sherkole camps [16] (Fig 1). Healthcare services are provided by international NGOs, UN agencies, and local authorities, focusing on primary care, maternal health, and communicable diseases. Despite these efforts, the service has faced some challenges, such as resource limitations and high demand, may still affect access to comprehensive care to detect and treat VL. These camps have ecological conditions conducive to sandfly survival, increasing the risk of VL transmission [13] with prior cases reported to the Ethiopian Ministry of Health [17].

### Study design and period

A cross-sectional study was conducted to determine the prevalence of *Leishmania* infection in selected refugee camps in the Gambella and Benishangul Gumuz regional states of Ethiopia. The study was conducted from May to August 2023 in refugee camps located in these regions.

### Study population

The study participants were refugees (five years of age and older) living in the selected camps. Individuals residing in the camp for at least six months and willing to provide assent and/or written informed consent were included in the study. Individuals who were unable to speak, listen or assent and/or provide written informed consent and who declined to participate or provide blood samples were excluded from the study.

### Sample size determination

The sample size was estimated using a standard formula for household based cross-sectional studies, $Nh=\frac{\left(Z^{2}\right)2(p)(1-p)(DE)(k)}{\left(\text{P})(n)(e^{2}\right)}$, where Z = 1.96 (95% confidence), p = 0.35 (proportion with VL) [18], DE = 1.5 (design effect), k = 1.1 (non-response multiplier), P = 0.95 (target population proportion), n = 4.0 (household size), and e = 0.025 (margin of error). This yielded the minimum sample size of 607 households from selected refugee camps in each regional states. Data were collected from 563 households in Gambella and 660 households in Benishangul-Gumuz, total 1,223 participants, with one participant selected per household.

### Sampling procedure

The refugee camps in Gambella and Benishangul-Gumuz regions were purposely selected because of high concentration of refugee camps. Within each region, the refugee camps chosen were Kule and Terkidi in Gambella, and Tsore and Sherkole in Benishangul-Gumuz. A simple random sampling technique was applied at the camp level, minimize an unbiased selection process for identifying camps that represent the refugee population adequately. The sampling interval (k = 24) was calculated by dividing the total number of households by the desired sample size. The random starting point between 1 and 24 was determined by a lottery method and number was 9 chosen as starting point. From this point, every 24^th^ household was systematically selected to ensure a representative and unbiased sample across all camps. This ensures that each camp contributes a sample size proportionate to its household population. The number of households allocated to each camp was based on its population size (S1 Fig).

### Data collection tools

A structured questionnaire was prepared based on the existing forms [19–23]. Characteristics of each participant’s sociodemographic status (i.e., age, sex, place of residence, number of family members) and clinical information (e.g., the presence of fever, headache, hepatomegaly or splenomegaly) were gathered.

### Clinical sample collection and processing

Five milliliter blood was collected in EDTA tube (Shenzhen medicalet technology Co. Ltd, Shenzhen City, China) from each study participant (n = 1223). The whole blood samples were centrifuged at 5,000 X g for 10 minutes to separate the plasma and buffy coat [24] and was stored at −20°C to preserve cellular components, ensuring the quality of the sample for *Leishmania* DNA extraction and PCR amplification [25].

### Direct agglutination test (DAT)

The direct agglutination test (DAT) was carried out as previously described [26]. Briefly, two µl of plasma samples was mixed with 100 µl diluent. The sample was diluted from 1:50–1:51200. *Leishmania* negative and positive controls prepared in an Academic Medical Center (Amsterdam) were used. Freeze-dried antigen (FDA) containing *L. donovani* promastigotes were added in all 96 wells of the V-shaped microtiter plate (Thermo Fisher Scientific, USA). The results were read after overnight incubation. A DAT agglutination of ≥1:1600 was considered positive for *Leishmania* infection, while samples of ≤1:800 were consider negative [19].

### DNA extraction

DNA extraction was performed from 754 randomly selected samples due to logistical constraints, using Geneaid DNA isolation kit (GMB300, Geneaid Biotech Ltd) per the manufacturer’s protocol. The extracted DNA was eluted in 100 µl of elusion buffer. A negative extraction control (NEC) or nuclease free water was used to identify contamination and confirm the integrity of the extraction reagents and the procedural steps involved and treated as sample.

### *Leishmania* kDNA detection by real-time polymerase chain reaction

A rtPCR targeting minicircle kinetoplast DNA (kDNA) was employed for the detection of *Leishmania* parasites from the buffy coat samples. The primers used for the study were kDNA-CMF (CTTTTCTGGTCCTCCGGGTAGG) and kDNA-CMR (CCACCCGGCCCTATTTTACAC CAA) [27]. The kDNA rtPCR was conducted as previously described [28]. Briefly, a 25 μl reaction volume was prepared with 1 × HotStarTaq Master mix (Qiagen, Venlo, The Netherlands), 0.6 μM of reverse and forward primers and 0.4 μM of the probe (Integrated DNA Technologies, Leuven, Belgium), 0.1 mg/mL bovine serum albumin (Roche, Vilvoorde, Belgium) and 5 μl of DNA template. The rtPCR was performed using QuantStudio 5 real-time PCR system (Catalogue: A28568, Thermo Fisher). During each run, the genomic DNA extracted from the *Leishmania* positive culture and the non-template control (NTC) were tested and used as a positive and negative control, respectively. Samples with cycle threshold (Ct) values between 12 and 40 with a sigmoidal amplification curve were considered positive for *Leishmania* infection [29].

### Data quality assurance

To ensure data quality, several measures were implemented, including the development of appropriate data collection tools and the provision of training for data collectors, laboratory professionals, clinicians and local guide before commencement of data collection. During the data collection process, questionnaires were reviewed daily for completeness, accuracy, and consistency. Blood sample collected, processed and diagnosed in laboratories and well experience laboratory experts. Quality control measures were applied for both DAT and rtPCR tests, utilizing positive and negative controls, and strict adherence to standard operating procedures was maintained throughout.

### Statistical analysis

The data were initially entered into Epi Info version 7.2 (Centers for Disease Control and Prevention, Atlanta, GA, USA), exported to Excel for data cleaning, then integrated into SPSS version 23.0 (SPSS Inc., Chicago, IL, USA) for analysis. Continuous variables were summarized with median and inter-quartile range (IQR) and categorical variables with numbers and proportions. Chi-square was used to assess the proportion differences among categorical variables. Association between the socio demography and clinical symptoms of the study participants with *Leishmania* positive participants were assessed by binary logistic regression. A multivariate logistic regression model was applied to variables with p < 0.20 in bivariate analysis. Adjusted ORs with a significance level of 0.05 were considered statistically significant for comparison.

## Results

### Sociodemographic characteristics

A total of 1223 participants were involved in the study from the planned 1214 individuals. In the Benishangul Gumuz region, 69.1% of respondents were females, while in the Gambella region, 58.26% were males. The majority of respondents in Benishangul Gumuz (61.8%) came from Sudan, while all participants in Gambella were from South Sudan. Education levels were 30.8% in Benishangul Gumuz and 41.2% in Gambella have no formal education. Over 63% of the refugees in both regions were married (Table 1). The median age of participants was 25 (IQR = 16 years) and 27 years (IQR = 25 years) for participants from Benishangul Gumuz and Gambella regions, respectively. The median length of stay in the camp was 8 years (IQR = 7 years) and 9 years (IQR = 4 years) participants from Benishangul Gumuz and Gambella refugee camps. The median family size of participants was 6 (IQR = 4) and 6 (IQR = 5) in refugee camps located in Benishangul Gumuz and Gambella regions, respectively.

**Table 1:** Socio demographic characteristics of study participants of selected refugee camps located in Gambella (N = 563) and Benishangul Gumuz (N = 660) regions, Ethiopia, 2023.

Categories		Camp location by region	
		Benishangul Gumuz N (%)	Gambella N (%)
Sex	Female	456 (69.1)	235 (41.7)
	Male	204 (30.9)	328 (58.3)
Age group in years	5-9	64 (9.7)	49 (8.7)
	10-19	202 (30.6)	141 (25.0)
	20-29	264 (40.0)	113 (20.1)
	30-39	71 (10.8)	121 (21.5)
	40-49	35 (5.3)	68 (12.1)
	50+	24 (3.6)	71 (12.6)
Camp Name	Kule	–	288 (51.2)
	Sherkole	220 (33.3)	–
	Terkidi	–	275 (48.9)
	Tsore	440 (66.7)	–
Country of Origin	South Sudan	252 (38.2)	563 (100.0)
	Sudan	408 (61.8)	0 (0.0)
Level of education	No formal education	203 (30.8)	232 (41.2)
	Primary school	342 (51.8)	232 (41.2)
	Secondary school	80 (12.1)	78 (13.9)
	Higher education	35 (5.3)	21 (3.7)
Marital Status	Married	428 (64.9)	358 (63.6)
	Single	232 (35.2)	205 (36.4)
Family Size	≤4	256 (38.8)	169 (30.0)
	>4	404 (61.2)	394 (70.0)

### Prevalence of *Leishmania* infection

Overall DAT positivity was 4.0% (95% CI: 3.0% to 5.3%) (49/1223). Most positive patients had a titer of 1:1600 or 1:3200; amongst the negative participants 275 still showed some agglutination (S2 Fig). Real-time PCR positivity was 10.3% (78/754) (95% CI: 8.3% to 12.7%). The cycle threshold (Ct) values were ranged from 30.9 to 39.9, with an average Ct value of 36.6.

The DAT positivity rates in the Benishangul Gumuz region were 5.9% in Tsore and 3.2% in Sherkole camps, while in the Gambella region, rates were 3.1% in Kule and 2.5% in Terkidi camps. For rtPCR, the highest positivity was observed in Tsore (14.6%) compared to Sherkole (3.2%, p < 0.05), whereas in Gambella, almost similar positivity rates were recorded in Kule (8.5%) and Terkidi (6.4%). Among the four refugee camps, rtPCR positivity was significantly higher for Tsore (14.6%, χ² = 21.4, p < 0.001), while no significant difference in DAT positivity was observed across camps in Benishangul Gumuz and Gambella (χ² = 6.6, p = 0.084).

Among individuals reporting specific symptoms, *Leishmania* DAT positivity rates varied as follows: those with fever had a DAT positivity rate of 6.0%, while individuals with chills and headache had also showed rates at 4.6% and 4.7%, respectively. Weakness was reported with a DAT positivity rate of 3.8% (Table 2). Among individuals who reported fever, 11.7% tested rtPCR positive for *Leishmania*. Those with chills, headache, and weakness had rtPCR a positivity rate of 8.8%, 7.3%, and 6.2%, respectively (Table 3). On the other hand, clinical symptoms were reported with the all of participants did not exhibit symptoms that strongly suggestive of VL including splenomegaly and hepatomegaly.

**Table 2:** Association between sociodemographic and clinical characteristics of participants and DAT positivity in selected refugee camps in Gambella and Benishangul Gumuz regions, Ethiopia, in 2023.

Category		Tested (%)	DAT_Pos (%)	OR (95%CI)	P value	AOR (95%CI)	p-value
Gender	Male	532 (43.5)	25 (4.7)	1.26 (0.71-2.23)	0.43	1.66 (0.90-3.07)	0.1
	Female	691 (56.5)	24 (3.5)	1		1	
Age Group	5-9	83 6.8)	6 (7.2)	1			
	10-19	310 (25.3)	10 (3.2)	0.536 (0.19-1.51)	0.24		
	20-29	377 (30.8)	11 (2.9)	0.536 (0.19-1.48)	0.23		
	30-39	219 (17.9)	9 (4.1)	0.877 (0.30-2.53)	0.81		
	40-49	122 (10.0)	8 (6.6)	1.50 (0.50-4.48)2	0.47		
	50+	112 (9.2)	5 (4.5)	0.991 (0.29-3.35)	0.99		
Region	Benishangul Gumuz	660 (54.0)	33 (5.0)	1.80 (0.98-3.31)	0.06	3.67 (1.57-8.59)	0.003
	Gambella	563 (46.0)	16 (2.8)	1		1	
Camp name	Kule	288 (23.5)	9 (3.1)	1		1	
	Terkidi	275 (22.5)	7 (2.5)	1.02 (0.37-2.78)	0.97	0.93 (0.35-2.48)	0.89
	Tsore	440 (36.0)	26 (5.9)	0.81 (0.30-2.21)	0.68	0.76 (0.27-2.13)	0.6
	Sherkole	220 (18.0)	7 (3.2)	1.95 (0.90-4.22)	0.09		
Country of Origin	South Sudan	815 (66.6)	37 (4.5)	1.57 (0.81-3.04)	0.18	2.87 (1.26-6.50)	0.012
	Sudan	408 (33.4)	12 (2.9)	1		1	
Education level	No formal education	435 (35.6)	26 (6.0)	1.72 (0.40-7.43)	0.47		
	Grade 1–8	574 (46.9)	13 (2.3)	0.63 (0.14-2.85)	0.54		
	Grade 9–12	158 (12.9)	8 (5.1)	1.44 (0.30-7.00)	0.65		
	College and above	56 (4.6)	2 (3.6)	1			
Marital Status	Married	786 (64.3)	33 (4.2)	1.15 (0.63-2.12)	0.65		
	Single	437 (35.7)	16 (3.7)	1			
Family size Cat	≤4	723 (59.1)	18 (2.5)	1	0.77		
	>4	500 (40.9)	31 (6.2)	0.91 (0.51-1.65)			
Fever	No	1005 (82.2)	36 (3.6)	1		1	
	Yes	218 (17.8)	13 (6.0)	1.71 (0.89-3.28)	0.11	2.08 (1.01-4.28)	0.047
Chills	No	1093 (89.4)	43 (3.9)	1			
	Yes	130 (10.6)	6 (4.6)	1.18 (0.49-2.83)	0.71		
Headache	No	1009 (82.5)	39 (3.9)	1			
	Yes	214 (17.5)	10 (4.7)	1.22 (0.60-2.48)	0.59		
Weakness	No	1065 (87.1)	43 (4.0)	1			
	Yes	158 (12.9)	6 (3.8)	0.94 (0.39-2.24)	0.87		

DAT: direct agglutination test, DAT_Pos = *Leishmania* positive by DAT, AOR: Adjusted Odds Ratio, CI: Confidence Interval

**Table 3:** Association between sociodemographic and clinical characteristics and participants’ rtPCR positivity in selected refugee camps in Gambella and Benishangul Gumuz regions, Ethiopia, in 2023.

Category		PCR Tested (%)	PCR Pos (%)	OR (95%CI)	p value	AOR (95%CI)	p value
Gender	Male	258 (34.2)	25 (9.7)	0.90 (0.54-1.48)	0.67		
	Female	496 (65.8)	53 (10.7)	1			
Age Group in years	5-9	72 (9.5)	9 (12.5)	1			
	10-19	224 (29.7)	22 (9.8)	0.76 (0.33-1.74)	0.52		
	20-29	284 (37.7)	26 (9.2)	0.71 (0.32-1.58)	0.40		
	30-39	88 (11.7)	8 (9.1)	0.70 (0.26-1.92)	0.49		
	40-49	48 (6.4)	6 (12.5)	1.00 (0.33-3.02)	1.00		
	50+	38 (5.0)	7 (18.4)	1.58 (0.54-4.64)	0.41		
Region	Benishangul Gumuz	648 (85.9)	70 (10.8)	1.48 (0.69-3.18)	0.31	1.84 (0.68-4.98)	0.23
	Gambella	106 (14.4)	8 (7.5)	1		1	
Camp name	Kule	59 (7.8)	5 (8.5)	0.54 (0.21-1.41)	0.21	0.54 (0.20-1.47)	0.23
	Terkidi	47 (6.2)	3 (6.4)	0.40 (0.12-1.32)	0.13	0.48 (0.14-1.70)	0.26
	Sherkole	217 (28.8)	7 (3.2)	0.20 (0.09-0.44)	<0.001	0.19 (0.08-0.45)	<0.001
	Tsore	431 (57.2)	63 (14.6)	1		1	
Country of Origin	South Sudan	357 (47.3)	42 (11.8)	1.35 (0.84-2.15)	0.22	0.98 (0.58-1.66)	0.94
	Sudan	397 (52.7)	36 (9.1)	1		1	
Education level	No formal education	241 (32.0)	30 (12.4)	5.40 (0.72-40.81)	0.10	2.49 (0.31-19.96)	0.39
	Grade 1–8	378 (50.1)	37 (9.8)	4.12 (0.55-30.91)	0.17	1.91 (0.24-15.03)	0.54
	Grade 9–12	96 (12.7)	10 (10.4)	4.42 (0.55-35.75)	0.16	2.44 (0.29-20.64)	0.41
	College and above	39 (5.2)	1 (2.6)	1		1	
Marital Status	Married	489 (64.9)	52 (10.6)	1.09 (0.66-1.78)	0.75		
	Single	265 (35.1)	26 (9.8)	1			
Fever	No	660 (87.5)	67 (10.2)	1			
	Yes	94 (12.5)	11 (11.7)	1.17 (0.60-2.31)	0.64		
Chills	No	686 (91.0)	72 (10.5)	1			
	Yes	68 (9.0)	6 (8.8)	0.83 (0.35-1.98)	0.67		
Headache	No	644 (85.4)	70 (10.9)	1		1	
	Yes	110 (14.6)	8 (7.3)	0.64 (0.30-1.38)	0.26	0.63 (0.23-1.73)	0.37
Weakness	No	673 (89.3)	73 (10.8)	1		1	
	Yes	81 (10.7)	5 (6.2)	0.54 (0.21-1.38)	0.19	0.56 (0.23-1.73)	0.35

rtPCR: real time polymerase chain reaction, AOR: Adjusted Odds Ratio, CI: Confidence Interval

**Fig 2:**
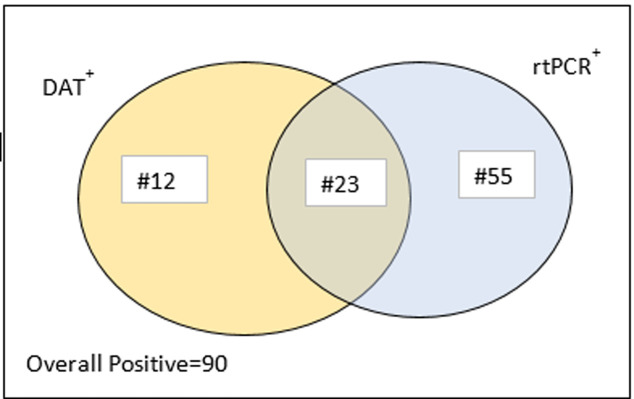
Venn diagram of DAT and rtPCR Positivity (n = 90) [DAT+ (Direct Agglutination Test Positive): represented by the yellow circle; rtPCR+ (Real-time PCR Positive): represented by the blue circle and the intersection (23 cases) indicates samples that tested positive in both methods].

Out of 754 individuals tested with both PCR and DAT, overall, 90 participants were positive for *Leishmania* infection. This represents an overall positivity rate of 11.9% (90/754) (95%CI: 9.7% -14.1%). Twelve samples tested positive only with DAT, 55 tested positive only with rtPCR, and 23 tested positives with both methods, for a total of 90 positive results overall reported. As illustrated in the Venn diagram that, while some samples showed agreement between the two tests, rtPCR identified substantially more positive cases than DAT (Fig 2).

### Factors related to *Leishmania* positivity

Sociodemographic and clinical factors related to *Leishmania* DAT positivity are shown in Table 2. In multivariate analysis, region was significantly related to DAT positivity, with participants from Benishangul Gumuz that had 3.67 higher odds of being positive [AOR: 3.67 (95%CI; 1.57-8.59); p = 0.003]. Highest odds of DAT positivity were found in South Sudanese participants, with an AOR of 2.87 (1.26-6.50) compared to Sudanese participants (p = 0.012). Moreover, participants with fever had 2.08 more times higher DAT positivity rate than with no fever [AOR: 2.08 (1.01-4.28); p = 0.047].

Sociodemographic and clinical assessment factors related to *Leishmania rtPCR* positivity with characteristics are shown in Table 3. In adjusted analysis, refugee camp was significantly related to *Leishmania* rtPCR positivity, with participants from Sherkole refugee camp showed that 81% more protective than living in Tsore camps in Benishangul Gumuz region [AOR: 0.19 (95%CI; 0.08-0.45); p < 0.0001].

## Discussion

Visceral leishmaniasis is a major public health concern in East Africa, including refugee camps in the region. In Ethiopia; the Gambella and Benishangul Gumuz regional states host many refugees living in conditions that favor *Leishmania* transmission [12]. The burden of *Leishmania* infection remains uninvestigated in refugee camps. This study aimed to determine *Leishmania* infection prevalence in these vulnerable populations using serological (DAT) and molecular (rtPCR) methods.

The prevalence rate was 4.0% and 10.3% as determined by DAT and rtPCR, respectively. The study participants did not report the typical VL symptoms in the data collection period. The prevalence and absence of clinical symptoms we reported highlights the asymptomatic reservoirs could be sustaining VL transmission in the study area. This necessitates implementation of active case detection, community-wide screening, rigorous vector control measures, and targeted public awareness campaigns to effectively combat and halt the transmission and progression of VL.

The overall DAT positivity rate in the current study was 4.0%. There are studies that showed higher DAT positivity rates in other regions of Ethiopia, including 18.1% in Gambella region among refugees in 1996 [30], 9.6% in southeastern Ethiopia [31] and 12.5% among agricultural migrant laborers in northwest Ethiopia [32]. In addition, a household-based survey in eastern Sudan showed serological positivity rates of 8.9% [33], and 13% in another study in southwest Sudan [34]. The lower DAT positivity rate in our study may be attributed to the refugees’ prolonged stay in camps (average of nine years) in Benishangul Gumuz and Gambella, with displaced from high-risk areas in Sudan and South Sudan to relatively lower-risk regions in Ethiopia. Although these regions have ecological conditions favorable for VL transmission [12,13], they are not known as endemic areas in Ethiopia. On the other hand there are other studies that showed similar DAT positivity rate findings; in Benishangul Gumuz [35,36] and among pastoralist communities in Borena zone Oromia regional states of Ethiopia [37], as well as in Gedarif state in Sudan [38]. These reports suggest comparable levels of exposure to *Leishmania* in certain endemic settings but emphasizing the sustained need for targeted interventions and surveillance in these areas.

There was no difference in the positivity rate between gender by DAT and rtPCR for *Leishmania* infection. In contrast to our findings, previous reports showed that L*eishmania* infection was higher in males in studies conducted in Ethiopia [35,36] and Sudan [39]. On the other hand, our data were in line with community based reports from the Somali region, South Omo zone [19,22] and Sudan [40]. The lack of gender-based differences in *Leishmania* infection in our study is likely a reflection of the unique sociocultural and environmental conditions in refugee camps, which create a more uniform risk setting compared to resident populations. The distribution of *Leishmania* infection among refugee camps as determined by both DAT and rtPCR was similar among age groups in the current study. Similar findings were reported from the Somali region in Ethiopia [22]. However, Abera et al reported that the prevalence of *Leishmania* infection gradually increased with age [35]. Susceptibility of gender and age to *Leishmania* infection may differ across societies based on prevailing patterns of household labor division [22] and living conditions specific for individuals living in refugee camps.

In the current study, the region of residence did not significantly predict the higher proportion of rtPCR positivity. On the other hand, the rtPCR positivity rate varied significantly among refugee camps in Benishangul Gumuz and Gambella regional states; Sherkole camp had the lowest positivity rate (3.2%) while other camps did not show significant differences. The DAT positivity rate showed significant variation, with refugee camps in the Benishangul Gumuz regional state facing a higher risk compared to those in the Gambella regional state. While these regions are not well known VL endemic foci, there is only one report on assessment of VL from 1996 in the Gambella [30] and few from the Benishangul Gumuz regional states [35,36,41].

Camp location may influence DAT positivity rates, with lower rates observed in Sherkole camp. In another study conducted among residents of Kumuruk district, which is adjacent to Homosha district, where these refugee camps located; reported an LST prevalence of 14.1% (27/191) with one infected dog in the community out of 36 tested (2.8%) [36]. On the other hand, no *Leishmania* infection was reported from the same study in other districts (Sherkole and Bambasi). These districts are not in immediate proximity to the current study area. Variations in *Leishmania* infection rates between bordering and non-bordering districts near the camp indicated that proximity to these districts significantly influences leishmaniasis transmission risk. Moreover, further serological surveys should be conducted in neighboring local populations to better evaluate whether refugees represent a risk of *L. donovani* introduction in these regions and/or if the living conditions in refugee camps are facilitating *Leishmania* transmission within this specific population.

The current study showed no difference in risk of acquiring *Leishmania* infection among individuals who lived in the camp for varying durations. The lack of association between camp duration and *Leishmania* infection risk suggests continuous exposure to the parasite regardless of length of stay in the camp. Other possible factors like vector density, personal protection measures, or immunity levels of the participants may play a more significant role in infection risk than length of stay. Nationality wise, a higher DAT positivity was observed in individuals from South Sudan, compared to those from Sudan. This could be due to most of the study participants came from the Jonglei and Upper Nile states of South Sudan. These regions reported the highest percentage of VL cases among 10 states in South Sudan [42].

Unlike the current finding, previous studies have shown that fever, splenomegaly, and hepatomegaly are major signs of VL in Sudan [39,43], South Sudan [44] and Ethiopia [45]. However, in the current study, only fever was associated with *Leishmania* infection. These earlier studies were conducted on diseased individuals, while the current report was based on community-level studies. Asymptomatic individuals tend to have a lower parasite load, which may not trigger noticeable clinical symptoms. Variations in immune responses to *Leishmania* infection can lead to different clinical outcomes, with some individuals effectively controlling the parasite and limiting the disease to fever, while others may develop more extensive organ involvement. Furthermore, in this study, all *Leishmania*-infected participants were asymptomatic for VL. However, HIV infection is known factor to significantly increase the risk of progression from asymptomatic infection to VL [28]. Due to logistical constraints, we were unable to screen participants for HIV status. We recommend further research on the epidemiology of HIV-VL co-infection in this population.

Since the distribution of sand flies within the camps has not yet been studied, and no clinically suspected *Leishmania*-infected individuals have been identified, our ability to directly assess the potential for *Leishmania* transmission within the camps was limited. However, our study provides insight into the distribution of the disease in the refugee camps we studied. Additionally, previous reports [30,36,46] and VL risk assessments in Ethiopia have classified the Gambella and Benishangul Gumuz regions as high-risk areas for VL transmission [12], suggesting that the camps may also be at risk for VL transmission.

The current results suggest that rtPCR detected 78 positive *Leishmania* infections compared to the 35 identified by DAT. The presence of 12 DAT-positive but rtPCR-negative cases could be attributed to persistent antibodies from past infections or false negatives in rtPCR due to low parasite DNA loads, as supported by studies indicating that serological tests can remain positive long after parasite clearance [27,47]. The detection ability variation among these two testing methods has also been corroborated by other studies conducted in Ethiopia among asymptomatic individuals [27,48,49]. However, the current report contradict with the study of cohorts of HIV infected subjects in known endemic areas from Ethiopia [28]. This variation could be the difference in study subjects (people living with HIV vs HIV status not mentioned). The discrepancy between the two test results suggests that combining both methods may enhance diagnostic accuracy. Public health strategies should incorporate rtPCR for confirmatory testing while using DAT for initial screening to improve case detection and disease control efforts [27,50]. Ultimately, integrating molecular diagnostics with serological methods is essential to strengthen VL surveillance and management programs. Even though the report indicated that detecting very low blood parasitemia is not consistent parameter for determining *Leishmania* infections [51], its usefulness can be enhanced by optimizing laboratory conditions, such as strict contamination monitoring, the inclusion of known positive and negative controls, and the use of extraction controls. By implementing these measures, as we did in our laboratory, the reliability and accuracy of the final results can be improved.

### Strengths and Limitations of the study

This study on the prevalence of *Leishmania* infection in refugee camps in Gambella and Benishangul-Gumuz, Ethiopia, offers significant strengths by utilizing both serological and molecular diagnostics to improve detection and by focusing on a high-risk, underserved refugee population. Our findings can potentially guide health interventions in leishmaniasis hotspots, providing critical insights for targeted interventions. There are several limitations to acknowledge. The significant disparity in sample sizes between the DAT and rtPCR presents a critical challenge to this study. DAT’s typical limitations, including lower sensitivity and specificity, can lead to misclassification of cases, potentially skewing prevalence estimates and underestimating the true disease burden, particularly in asymptomatic individuals or those with low parasite loads. The underrepresentation of rtPCR results, a more sensitive method for detecting early-stage and subclinical infections, may limit the study’s ability to accurately capture the true disease dynamics. This imbalance hinders the analysis using both testing tools, weakening conclusions regarding their diagnostic accuracy and potentially introducing a bias towards DAT outcomes. We did not study the genetic diversity or the presence of specific *Leishmania* species, and cannot differentiate between past and current infections or track changes in infection status over time. Despite these limitations, the study provides valuable public health information for targeted disease control in vulnerable populations. Addressing these limitations in future research could further strengthen the findings and applicability of such studies in similar settings.

## Conclusion and recommendation

*Leishmania* infection was prevalent in refugee camps in Gambella and Benishangul Gumuz regions, with detection rates of 4.0% using DAT and 10.7% using rtPCR. *Leishmania* infection was reported in all studied camps, although at varied proportions, with a noteworthy presence of asymptomatic cases and low parasite loads. These findings underscore the necessity for region-specific interventions, particularly given the higher positivity rates in the Benishangul Gumuz region and among South Sudanese refugees. This highlights the importance of tailored strategies, including active case detection, community-based screening, vector control measures, to effectively manage and reduce VL transmission within these high-risk populations. We additionally recommend assessing the presence of the vector sand-fly in both regions around the camp area. Continuous monitoring and comprehensive healthcare support are vital for effectively managing and mitigating the impact of leishmaniasis among this vulnerable population.

## Supporting information

S1 Fig Schematic presentation of sampling procedure (n = 1223) in this cross-sectional study. HH household: This figure depicted the selection process of refugee camps and households in two regions, Gambella and Benishangul Gumuz, using different sampling techniques. Regions were purposely selected followed by a simple random sampling technique to select camps. Finally, a systematic sampling technique with household (HH) proportion allocation was applied to determine the number of surveyed households in each camp.
(TIF)

S2 Fig Distribution of anti-*Leishmania* antibody agglutination among selected refugee camps participants in Gambella and Benishangul Gumuz regions, 2023: This figure presents the distribution of Direct Agglutination Test (DAT) results based on agglutination titers. The x-axis represents the number of samples, while the y-axis categorizes the results into DAT-positive and DAT-negative groups based on different dilution levels. Samples without agglutination are also indicated. Higher dilution ratios (e.g., 1:25600, 1:12800) represent stronger positive reactions, while lower dilution ratios and “No agglutination” indicate negative results.
(TIF)
