# Supplementary figures and images for: Prevalence of Leishmania infection in refugee camps: A serological and molecular study in Gambella and Benishangul-Gumuz, Ethiopia
Source: PLoS Negl Trop Dis. 2025 Jul 8;19(7):e0013280. doi: 10.1371/journal.pntd.0013280 (PMC12262885; doi:10.1371/journal.pntd.0013280)

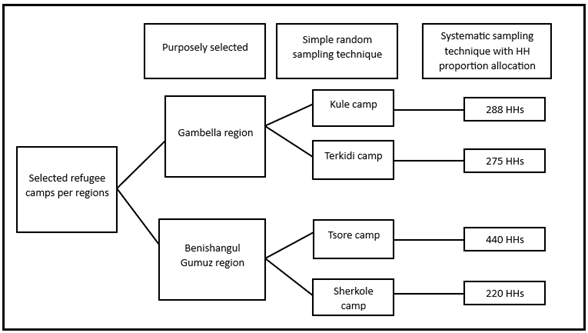

Supplement: S1 Fig — Schematic presentation of sampling procedure (n = 1223) in this cross-sectional study. HH household: This figure depicted the selection process of refugee camps and households in two regions, Gambella and Benishangul Gumuz, using different sampling techniques. Regions were purposely selected followed by a simple random sampling technique to select camps. Finally, a systematic sampling technique with household (HH) proportion allocation was applied to determine the number of surveyed households in each camp. (TIF) [file pntd.0013280.s001.tif]

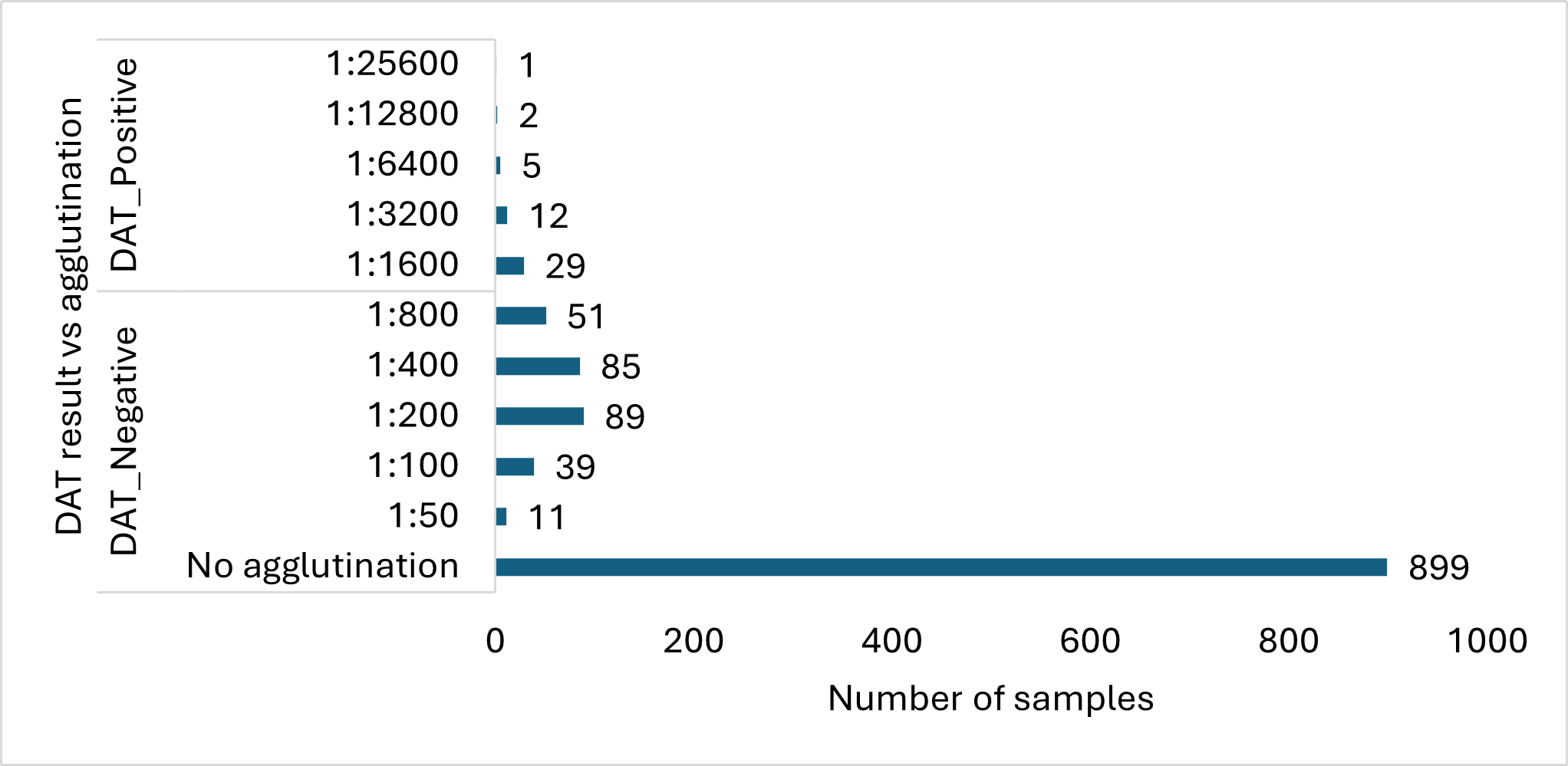

Supplement: S2 Fig — The x-axis represents the number of samples, while the y-axis categorizes the results into DAT-positive and DAT-negative groups based on different dilution levels. Samples without agglutination are also indicated. Higher dilution ratios (e.g., 1:25600, 1:12800) represent stronger positive reactions, while lower dilution ratios and “No agglutination” indicate negative results. (TIF) [file pntd.0013280.s002.tif]
